# Use of Computed Tomography, Ultrasound, and Vaginoscopy in the Evaluation of a Bilobed Vaginal Müllerian Cyst in a Neutered Female Labrador Retriever

**DOI:** 10.1155/crve/5706632

**Published:** 2025-09-02

**Authors:** Ying-Ying Lo, Emmanuel Topie, Stéphanie Moreau, Dimitri Leperlier, Anne-Sophie Bedu

**Affiliations:** ^1^Centre Hospitalier Vétérinaire AniCura Pommery, Reims, France; ^2^Anirepro, Reims, France; ^3^Cerba Vet, Massy, France

## Abstract

An 11-year-old neutered female Labrador was presented with dyschezia and dysuria associated with a large perineal mass that had been present for 2 years. Computed tomography (CT) revealed a large bilobed, cavitated mass localized ventrally to the rectum and dorsally to the urethra at the level of the vagina, with hypoattenuating contents and a contrast-enhancing peripheral wall. Surgical excision was performed and confirmed the cystic nature of the mass. Histopathological findings were compatible with a benign vaginal cyst, most likely originating from the Müllerian or paramesonephric ducts. The CT provided relevant information for surgical planning and enabled accurate assessment of the mass's location, extent, and relationship with adjacent structures.

**Summary**

Vaginal cysts are an uncommon condition in dogs. Histological examination can provide information on their origin. Computed tomography is useful for diagnosis, including assessment of size, local extension, and surgical planning.

## 1. Introduction

Vaginal cysts are commonly reported in humans and sporadically in cattle and dogs. They may be congenital or acquired and either incidental or associated with clinical signs. The most frequent clinical signs are dysuria and/or dyschezia [1]. In cattle, reproductive issues can also occur [2]. Vaginal cysts are usually classified based on histopathological analysis [3].

Only a few cases of congenital vaginal cysts have been reported in dogs [1, 4]. In humans, Müllerian cysts are the most common [1]. They originate from the paramesonephric ducts and are typically lined with mucinous epithelial cells [3]. Gartner duct cysts arise from remnants of the mesonephric ducts and are lined by nonmucinous, low-columnar to cuboidal epithelium [3].

Acquired vaginal cysts are rare in dogs and usually represent epidermal inclusion cysts [1], which develop following a trauma or surgery. These cysts are lined by a squamous epithelium and contain keratinous debris [3]. A few reports have also described Bartholin's [5] or clitoral cysts [6], though these are anecdotal and generally arise from the external genital tract rather than the vagina itself.

The cyst's location can help to determine its origin [3]. Gartner cysts are typically found on the lateral walls of the anterior vagina and are thus intraperitoneal. Müllerian cysts can occur anywhere along the genital tract, while inclusion cysts tend to develop at sites of prior trauma or surgery.

Ultrasound, CT, and/or magnetic resonance imaging (MRI) [1, 4, 7] are valuable tools to precisely localize the cyst and guide treatment planning.

We report here the CT evaluation of a large bilobed Müllerian vaginal cyst causing dyschezia and dysuria in a neutered female dog.

## 2. Case Presentation

An 11-year-old neutered female Labrador retriever was referred to the Centre Hospitalier Vétérinaire Pommery (Reims, France) for evaluation of a perineal mass. The lesion had been present for 2 years and was associated with dyschezia and dysuria. A vaginal origin was initially suspected by the referring veterinarian, and an ovariohysterectomy had been performed the previous year in an attempt to limit its growth. Nonsteroidal anti-inflammatory drugs and antibiotics were prescribed but were ineffective.

On physical examination, a firm, painless, 15 cm mass deforming the perineal area was palpated. Vaginal inspection confirmed the presence of an intraluminal vaginal mass. Routine bloodwork, including serum biochemistry and complete blood count, was unremarkable.

**Figure 1:**
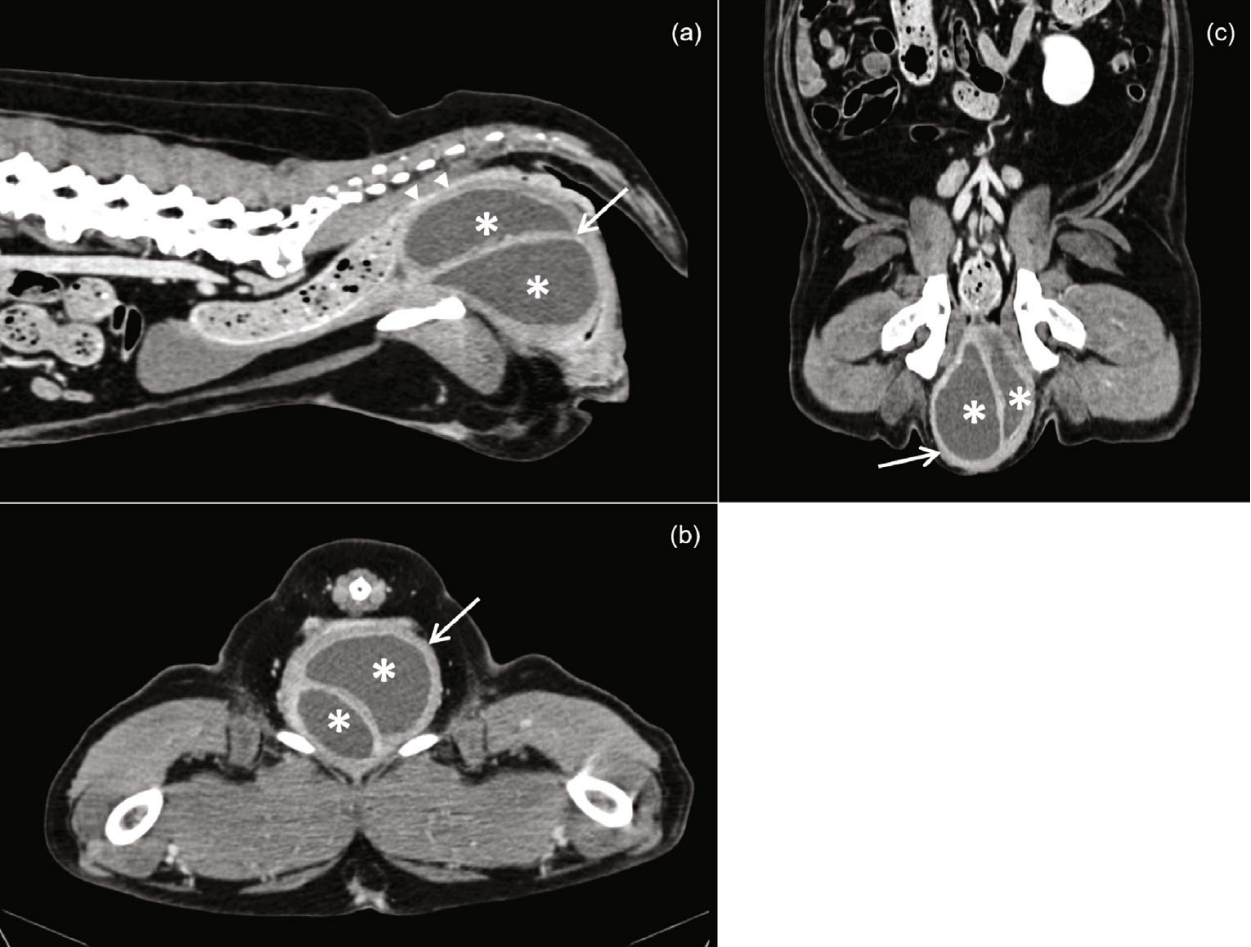
Postcontrast enhanced CT (a) sagittal, (b) transverse, and (c) dorsal images of the caudal abdomen and perineum displayed in soft tissue window. A large cystic fluid-attenuating (20–25 HU), oval-shaped, septated structure (∗) bounded by a thick enhanced wall (thin arrow) is observed in the area of the vagina. Notice the deformed perineum and the dorsal displacement and associated compression of the rectum (arrowheads).

Abdominal CT including the pelvic and perineal regions was performed under general anesthesia to assess the mass and its relationship to adjacent structures (Figure 1). Anesthesia was induced with diazepam (0.2 mg/kg IV) and propofol titrated to effect (4 mg/kg IV) and maintained with isoflurane. The dog was placed in sternal recumbency, and images were acquired using a 64-slice CT scanner (Siemens SOMATOM go.All; Erlangen, Germany) at 110 KVp and 130 mAs before and after intravenous administration of iohexol at the dose of 600 mg iodine/kg (Omnipaque 350 mg I/mL; GE Healthcare, Amsterdam, the Netherlands). Multiplanar reformatted images in sagittal and dorsal planes were generated using a soft tissue algorithm (window width: 350; window level: 40) and reviewed using medical imaging analysis software (Osirix MD v12.5.3, Bernex, Switzerland).

The mass was located ventral to the rectum and dorsal to the urethra. It demonstrated a large, homogeneous, hypoattenuating, fluid-filled content (20–25 HU) surrounded by a thick, regular, hyperattenuating wall (Figure 1). Internal septation separated the mass into two adjacent cavities. Postcontrast images showed homogeneous enhancement of the wall, while the fluid content remained unchanged (Figure 1). The mass caused significant dorsal displacement and compression of the rectum. The urethra could not be clearly delineated from the lesion. Mild enlargement of the hypogastric and medial iliac lymph nodes was noted, consistent with reactive adenitis. Other abdominal structures appeared normal.

**Figure 2:**
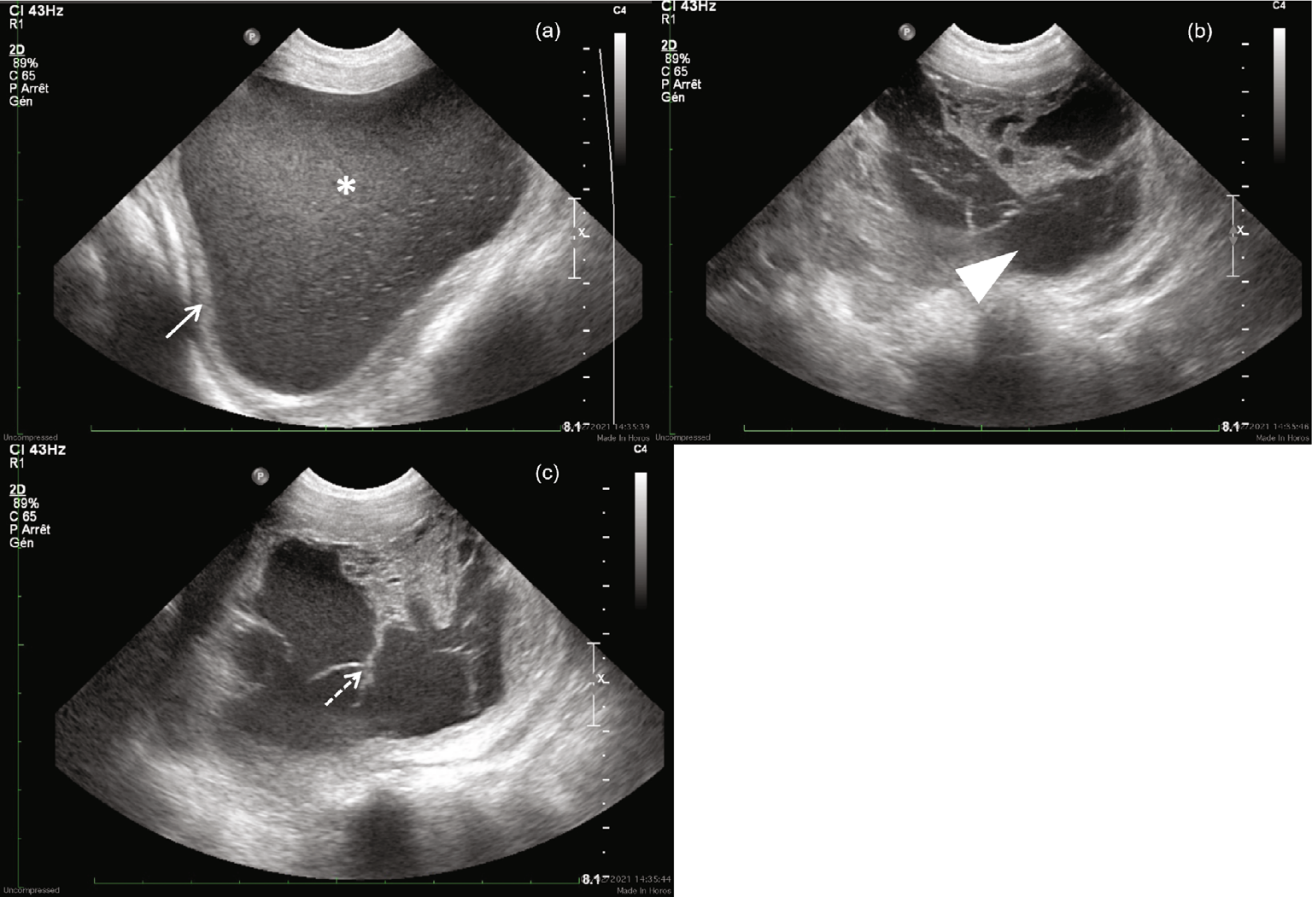
Ultrasound images of the cyst visible in the perineal area. The cyst is lined with a (a) thick hyperechoic wall (arrow) and demonstrates (b) multiloculated cavities (arrowhead) separated by (c) thin hyperechoic septations (dotted arrow). The fluid is echogenic and particulate (∗).

An abdominal ultrasound was subsequently performed using a 5–8 MHz microconvex probe (Philips iU22; Amsterdam, the Netherlands). It confirmed the cystic nature of the lesion, revealing fluid-filled cavities surrounded by thick, regular, hyperechoic walls (Figure 2). The fluid was slightly heterogeneous, with floating hyperechoic foci in one cavity and more organized, nonvascularized echogenic material in the other.

Vaginoscopy provided further evaluation. It confirmed the intravaginal location and revealed a large space-occupying mass distorting the pelvic floor of the vagina, with a translucent wall and prominent vascularization. The urethral meatus was visualized and appeared normal. These findings were valuable for surgical planning.

Surgical episiotomy and excision of the cystic structures from the vaginal wall were performed. Approximately 45 mL of translucent white fluid was removed. The excised tissues were submitted for histopathological analysis. The dog was discharged 24 h later with amoxicillin/clavulanic acid (12.5 mg/kg orally, BID) for 10 days and meloxicam (0.1 mg/kg orally, SID) for 5 days. Recovery was uneventful, and at both 1-month and 6-month follow-ups, the dog showed complete resolution of dyschezia and dysuria with no complications.

**Figure 3:**
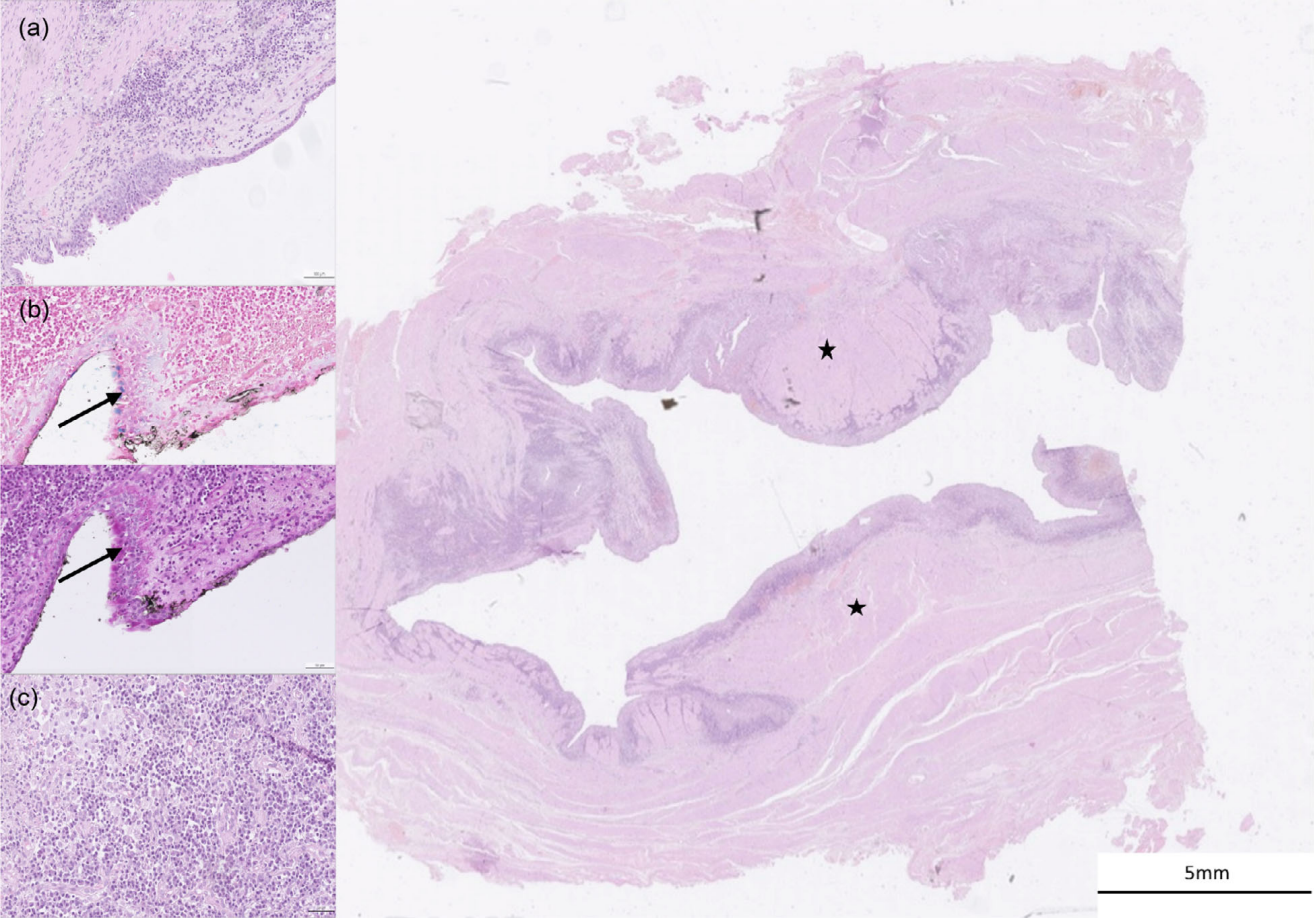
Histopathological examination of the resected tissue with H&E staining. (a) The structure is cystic and delineated by a thick muscular layer (black star) and lined by a uni- to pluristratified epithelium. (b) The epithelial cells are cubic, columnar to polygonal, and contain few cells with a PAS and Alcian blue vacuole in their cytoplasm (black arrows). (c) Mixed chronic inflammation and metaplasia of the epithelium are present with numerous plasma cells, lymphocytes, neutrophils, and macrophages under the mucosal layer.

Histopathological examination of the resected tissue with H&E staining revealed a cystic structure with a thick muscular wall lined by uni- to pluristratified epithelium (Figure 3). The epithelial cells were cuboidal to columnar or polygonal, with occasional PAS- and Alcian blue-positive vacuoles. Chronic inflammation was present in the cyst wall, with abundant plasma cells, lymphocytes, neutrophils, and macrophages beneath the mucosa, along with epithelial erosion and metaplasia. Based on these findings, a Müllerian duct cyst was considered the most likely diagnosis, although a Gartner duct cyst (mesonephric duct remnant) could not be completely ruled out.

## 3. Discussion

Vaginal cysts in mammals are fluid-filled structures covered by a thin wall. Congenital cysts usually arise from developmental anomalies and originate from various parts of the reproductive system. Diagnosis is usually based on imaging. The cyst's location and histopathological features help determine the type, based on the cellular composition of its wall [3].

While histopathology is often useful for differentiating cyst types, some overlap can occur [3, 8], especially in the presence of inflammation or metaplasia [3, 9, 10]. In our case, the absence of keratinizing squamous epithelium ruled out an epidermal inclusion cyst. A Gartner duct cyst was considered unlikely, as these are typically intraperitoneal [3, 10–13]. However, a few cases of very large Gartner duct cysts have been reported in women, with caudal extension leading to vaginal prolapse [14, 15]. In our patient, additional stains (PAS and Alcian blue) revealed mucin-containing cells, supporting the diagnosis of a Müllerian vaginal cyst rather than a Gartner duct cyst [16].

In some previously reported cases in bitches, fluid analysis revealed inflammation with neutrophils in two cases [11, 17], and one showed a positive bacterial culture [11]. In women, the presence of luminal keratin and squamous debris, along with a keratinized squamous epithelium lining, suggests an epidermal inclusion cyst [9, 10, 16, 18]. Müllerian cysts typically contain mucin, unlike Gartner duct cysts.

The unusual features of our case were the lobulated aspect of the cyst and its perineal location. The perineal deformation could initially have suggested a lesion originating outside the reproductive tract. This highlights the importance of including a vaginal examination alongside general clinical assessment when evaluating perineal swelling in female dogs.

Vaginal cysts are not systematically symptomatic in bitches [12], but when they enlarge, they can become clinically significant and may require surgical intervention [1]. Clinical signs such as dyschezia, tenesmus [1, 17], dysuria [1, 19], and pain [1] can occur due to compression of adjacent organs. In the present case, surgical resection was necessary to relieve compression and restore urinary and fecal patency.

Histopathology revealed inflammation within the cyst wall, suggesting an active process that could potentially lead to complications. A case of *Escherichia coli* infection has been reported in a similar context [11]. In women, abscess formation secondary to *Brucella melitensis* [20] has been associated with a Bartholin gland cyst. In our case, mild locoregional lymphadenopathy was consistent with reactive adenitis, likely related to local inflammation, with no evidence of any other complications.

Some vaginal cysts have been discovered incidentally during ovariohysterectomy [21], occasionally in association with conditions such as hydrometra or asymptomatic congenital malformations like unilateral renal agenesis [12]. These cysts were typically smaller, abdominal in location, and sometimes multiple [21]. Severe adhesions between the cyst and the urinary tract have also been reported [1], potentially complicating surgery and reinforcing the value of preoperative imaging.

In cattle, genital cysts are most often Bartholin's cysts, with clinical presentation including vaginal prolapse or a mass protruding from the vulva [22–24]. These can also lead to infertility or dystocia [2]. In women, vaginal cysts may cause pelvic pain [7], perineal deformation [14], dyspareunia [7], or recurrent urinary tract infections [25] and may be associated with malformations such as ureteral ectopia [26] or urethrovaginal fistulas [27]. No such abnormalities were observed on abdominal CT in our patient.

In this case, CT was selected as the first-line imaging modality to clarify the nature and extent of the mass prior to surgical intervention. While radiography, vaginal urethrography, and ultrasonography could have confirmed the cystic and vaginal nature of the lesion, they would not have provided sufficient detail regarding its pelvic or caudal extension [1, 12, 17]. In our case, CT clearly identified the cystic nature of the mass, confirmed by ultrasound, and allowed precise evaluation of its extent into the pelvic region, as well as its relationship to the urethra and rectum. It also revealed rectal compression and excluded any concurrent urinary or genital anomalies.

In women, MRI is the imaging modality of choice for evaluating vaginal masses [16, 28] due to its excellent soft tissue contrast and ability to assess the entire pelvis and surrounding soft tissues without ionizing radiation [14, 15]. Axial and coronal images allow accurate delineation of the vaginal anatomy and help differentiate vaginal lesions from those arising in the uterus or ovaries [7, 14] while also detecting associated malformations [26]. Although MRI has not been described for the evaluation of vaginal cysts in dogs, similar diagnostic value can be expected. In our case, CT was preferred over MRI for practical reasons. Indeed, MRI is more expensive and requires a longer acquisition time, with prolonged anesthesia. Moreover, CT allowed rapid scanning and staging of the abdomen and thorax. Finally, given the limited veterinary literature on MRI for vaginal cysts, a significantly higher diagnostic yield was unlikely.

In conclusion, this case illustrates that a perineal mass in a female dog can originate from the vagina. It underscores the importance of considering vaginal cysts in the differential diagnosis of perineal swelling and of performing a vaginal inspection. The combination of CT, ultrasound, and vaginoscopy provides comprehensive information about the lesion's nature, origin, and extent, as well as any associated malformations that are essential for surgical planning.

## Data Availability

Data sharing is not applicable to this article as no datasets were generated or analyzed during the current study.
